# Improved Functional Causal Likelihood-Based Causal Discovery Method for Diabetes Risk Factors

**DOI:** 10.1155/2021/5552085

**Published:** 2021-05-14

**Authors:** Xiue Gao, Wenxue Xie, Zumin Wang, Bo Chen, Shengbin Zhou

**Affiliations:** ^1^College of Information Engineering, Lingnan Normal University, Guangdong 524048, China; ^2^College of Information Engineering, Dalian University, Dalian 116622, China

## Abstract

Diabetes mellitus is a disease that has reached epidemic proportions globally in recent years. Consequently, the prevention and treatment of diabetes have become key social challenges. Most of the research on diabetes risk factors has focused on correlation analysis with little investigation into the causality of these risk factors. However, understanding the causality is also essential to preventing the disease. In this study, a causal discovery method for diabetes risk factors was developed based on an improved functional causal likelihood (IFCL) model. Firstly, the issue of excessive redundant and false edges in functional causal likelihood structures was resolved through the construction of an IFCL model using an adjustment threshold value. On this basis, an IFCL-based causal discovery algorithm was designed, and a simulation experiment was performed with the developed algorithm. The experimental results revealed that the causal structure generated using a dataset with a sample size of 2000 provided more information than that produced using a dataset with a sample size of 768. In addition, the causal structures obtained with the developed algorithm had fewer redundant and false edges. The following six causal relationships were identified: insulin→plasma glucose concentration, plasma glucose concentration→body mass index (BMI), triceps skin fold thickness→BMI and age, diastolic blood pressure→BMI, and number of times pregnant→age. Furthermore, the reasonableness of these causal relationships was investigated. The algorithm developed in this study enables the discovery of causal relationships among various diabetes risk factors and can serve as a reference for future causality studies on diabetes risk factors.

## 1. Introduction

With the steady increase in the number of diabetic patients worldwide, diabetes mellitus has become the third most serious threat to human health after cerebro-cardiovascular diseases and malignant tumours [1]. Diabetes is a chronic metabolic disorder that can be caused by a wide variety of risk factors. It leads to disturbances in fat and protein metabolism, resulting in chronic injury or failure of multiple organs [2]. Diabetes severely impacts human health and imposes a heavy burden on families and societies; hence, there is a pressing need for effective prevention and treatment of diabetes. The analysis of the relationships among various risk factors and between diabetes and risk factors is essential to elucidate the pathogenesis of diabetes and is a precondition for diabetes prevention and treatment. Previous research in China and other countries has largely focused on two areas: (1) the analysis of risk factors for diabetes onset and (2) the construction of prediction models for diabetes onset.

1. Research on the analysis of risk factors for diabetes onset primarily comprises two activities: the exploration of new risk factors and relationship analysis of risk factors. The investigation of new risk factors enables the discovery of potential factors for diabetes onset, which is beneficial for understanding diabetes aetiologies and may facilitate the effective prevention of diabetes. As the pathogenesis of diabetes involves multiple factors, the analysis of the relationships among these risk factors is particularly important and of practical and clinical significance. (i) Researchers have discovered many new risk factors of diagnostic and predictive significance. For instance, Fizelova et al. [3] found that the apolipoprotein B/LDL cholesterol ratio and apolipoprotein A1/HDL cholesterol ratio are the strongest predictors of the worsening of glycaemia and incidence of type 2 diabetes, respectively, in Finnish men. Lankinen et al. [4] identified plasma fatty acids as a potential predictor for glycaemia and a risk factor for type 2 diabetes mellitus (T2DM) in Finnish men. Further, Yazdanpanah et al. [5] found that glycated albumin (GA) provides more accurate diabetes diagnosis than glycated haemoglobin. Another study by Huang et al. [6] revealed that adiponectin (ADPN) combined with fibroblast growth factor 21 (FGF-21) and adipocyte fatty acid binding protein (A-FABP) are of great clinical significance in the early diagnosis and risk prediction of T2DM and could serve as key markers for the prediction of T2DM onset in high-risk populations. Bellia et al. [7] demonstrated the clinical usefulness of GA in the diagnosis of diabetes in a high-risk Caucasian population. In another study, Tatsukawa et al. [8] found that the risk of diabetes in the Japanese population was significantly positively correlated with trunk fat and significantly negatively correlated with leg fat. Li et al. [9] revealed that the age of alcohol onset and drinking duration are risk factors for T2DM. (ii) Studies on relationship among the various risk factors have provided a basis and direction for the investigation of potential aetiologies of diabetes. Zhao et al. [10] explored the correlations of trace elements in serum with serum glucose and body composition indicators in T2DM patients and concluded that the correction of trace element metabolism disorders in T2DM patients may be of great significance for diabetes treatment and the prevention of complications. Tillin et al. [11] revealed that branched chain and aromatic amino acids, particularly tyrosine, may be potential treatment targets for diabetes in South Asian populations. In addition, Cui and Feng [12] found that body mass index (BMI) is positively correlated with body fat percentage and abdominal-glute ratio, which indicates that body fat percentage may be clinically significant for diabetes diagnosis. Huang et al. [13] constructed a correlation network with biomarkers related to T2DM, which showed that the leptin system plays a key role in diabetes development. Meanwhile, Zhu et al. [14] studied the relationship between diabetes and body composition and found that visceral fat content, total fat content, total lean body mass, trunk lean mass, and limb lean mass are influencing factors of glycated haemoglobin. Therefore, glycaemic control in T2DM patients may be associated with lean body weight. Through Mendelian randomisation analysis, Liu et al. [15] found that there is a causal relationship between the genetically driven nonalcoholic fatty liver disease (NAFLD) and central obesity, both of which are risk factors for diabetes
2. Early research on diabetes prediction models mainly involved the use of statistical regression methods for model construction, with typical examples including a model developed by Chien et al. for predicting T2DM risk in the Taiwanese population [16], a prediction model for diabetes onset developed by Li et al. [17], a classification tree model for diabetes prediction in rural Chinese [18], a model for the prediction of T2DM risk in Japanese Americans [19], the Finnish Diabetes Risk Score tool [20], and a diabetes risk prediction model for a mixed African American and non-Hispanic white population [21]. In recent years, rapid developments in artificial intelligence techniques have led to the adoption of machine learning methods to construct diagnostic and predictive models of various diseases. Intelligent diagnosis and prediction methods for different diseases can be classified into two categories: one based on traditional single learner and the other based on multiple learners, such as the diabetes diagnosis method based on a single learner proposed by Rahman et al. [22] and the congestive heart failure diagnosis method based on multiple learners proposed by Isler et al. [23]. In the diagnosis and prediction of diabetes mellitus, the approach based on a single learner can provide satisfactory results with higher efficiency. For instance, Wang and Chen [24] utilised a support vector machine (SVM) with different kernel functions to construct prediction models for T2DM risk and found that the radial basis function-based SVM model provided the best predictive effects. Song et al. [25] and Chen et al. [26] reported the application of back-propagation neural network models to T2DM risk prediction. In addition, some researchers have improved the traditional single learner approach for better diagnosis and prediction. Erkaymaz et al. [27] found that Newman-Watts small-world feedforward neural networks have better accuracy in diagnosing diabetes, by comparing two different small-world feedforward neural networks. Geman et al. [28] used an adaptive neuro-fuzzy inference method to establish a diabetes classification and prediction system, which provided good classification and prediction accuracy. Further, several scholars have committed to exploring diabetes prediction methods based on multiple learners for better accuracy. For example, Liu et al. [29] developed a diabetes prediction model through the integration of SVM and the random forest (RF) technique and found that the integrated model provided superior classification performance compared with single classifiers. López et al. [30] used the RF technique to identify single-nucleotide polymorphisms in T2DM and to construct a decision-support tool for diabetes risk prediction. Wu et al. [31] used deep neural network and logistic regression models to predict gestational diabetes in the Chinese population, with better prediction performance that previous methods

Research on relationship among risk factors may enable the discovery of previously unknown physiological and pathological phenomena of diabetes, providing a theoretical basis for the elucidation of diabetes pathogenesis. However, existing studies on the relationships among risk factors mostly reflect the correlations rather than causality among these factors. Although diabetes prediction models are beneficial for diabetes prevention and early diagnosis, they are fundamentally statistical correlation models that do not reflect causality. Therefore, there is a pressing need for studies on the causality of diabetes risk factors, as the determination of the pathological and physiological causal relationships of diabetes is of great theoretical significance and could provide clinical guidance for diabetes prevention and treatment.

Randomised controlled trials (RCTs) [32] constitute a traditional method of causality discovery. However, substantial interventions are required for the experimental group in an RCT, which are costly and may entail ethical and moral violations. These issues can be avoided by using observational data-based causal discovery methods, but noise in the data may influence the effects of causal discovery algorithms. In situations with significant noise, functional causal likelihood- (FCL-) based algorithms [33] can effectively discover causal relationships. However, in the discovery of causal relationships among diabetes risk factors, numerous redundant and erroneous causal edges are generated when using these algorithms. To overcome this problem, we developed an improved functional causal likelihood- (IFCL-) based diabetes risk factor causal discovery algorithm to uncover causal relationships among diabetes risk factors. Our study is the first to use the causal discovery algorithm to explore the causal relationship between diabetes risk factors.

The contributions of the present study are as follows:

1. An IFCL model was developed by incorporating an adjustment threshold value *α*, which reduces the number of redundant and erroneous edges in the diabetes risk factor causal structures
2. An IFCL-based diabetes risk factor causal discovery algorithm was subsequently constructed and used to generate optimised diabetes risk factor causal structures
3. A simulation experiment was performed for comparative analysis of causal structures generated using different methods and sample sizes, and the significance of the identified causal relationships was assessed

The remainder of this paper is organised as follows.  Section 2 provides the details of the IFCL model and diabetes risk factor causal discovery algorithm.  Section 3 describes the experimental process and provides an analysis and discussion of the experimental results. Finally, Section 4 presents the study conclusions.

## 2. Materials and Methods

### 2.1. IFCL Model

The fundamental concepts of the FCL model are the assumption that the noise term is independent and is incorporated into the likelihood and that the likelihood over observational data is converted into the likelihood over the noise of the observational data and subsequently solved. Let {*X*_1_, *X*_2_, ⋯, *X*_*N*_} denote the variable set for diabetes risk factors, where *N* is the number of risk factor variables. *G* denotes the causal graph of the subset *X* = {*X*_1_, *X*_2_, ⋯, *X*_*n*_}, *P*(*X*_*i*_ = *x*) is the probability that *X*_*i*_ = *x*, and *P*(*X*_*i*_ | *P*_*i*_) indicates the probability of observations on *X*_*i*_ with conditions on the values of all its parents *P*_*i*_, with 1 ≤ *i* ≤ *n* ≤ *N*. Given that *G* satisfies the causal Markov condition [32, 34] and causal faithfulness condition [32], the joint distribution *P*(*X*) can be expressed as follows:

(1) $$\begin{array}{c} P\left(X\right)=\overset{n}{\underset{i=1}{\prod }}P\left(X_{i}\;|\;X_{P_{i}}\right), \end{array}$$

where *X*_*P*_*i*__ includes all parents of *X*_*i*_. Given a group of observational data $O=\left\{\vec{o_{1}},\vec{o_{2}},\cdots ,\vec{o_{j}},\cdots ,\vec{o_{m}}\right\}$, where $\vec{o_{j}}$ is an *n*-dimensional vector (i.e., $\vec{o_{j}}=\left(o_{j,1},o_{j,2},\cdots ,o_{j,n}\right)$, 1 ≤ *j* ≤ *m*), *o*_*j*,*P*_*i*__ can be used to denote the subvector of $\vec{o_{j}}$ containing the observational values of *X*_*P*_*i*__. By combining *P*(*X*) and *G*, the log-likelihood of the observational data can be expressed as follows:

(2) $$\begin{array}{c} L\left(G;O\right)=\overset{m}{\underset{j=1}{\sum }}\overset{n}{\underset{i=1}{\sum }}\log\left(P\left(X_{i}=o_{j,i}\;|\;X_{P_{i}}=o_{j,P_{i}}\right)\right). \end{array}$$

A search for causal networks by maximising the likelihood calculated using Equation (2) may not return true causality structures owing to the possible existence of different graphical structures providing exactly the same likelihood, which are known as Markov equivalence classes. To overcome the issues associated with Markov equivalence classes, it is necessary to introduce the concepts of causal function and noise.

**Figure 1:**
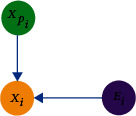
Partial causal structure consisting of *X*_*P*_*i*__ and *X*_*i*_.

Figure 1 shows a partial causal structure, with *E*_*i*_ and *X*_*P*_*i*__ denoting the randomised noise corresponding to *X*_*i*_ and the causal variable of *X*_*i*_, respectively. An additive noise model *X*_*i*_ = *F*_*i*_(*X*_*P*_*i*__) + *E*_*i*_ is adopted as the causal mechanism, with *F*_*i*_ being the causal function of *X*_*i*_ and the randomised noise variable *E*_*i*_ being independent of the causal variable *X*_*P*_*i*__. Therefore, the following equation can be derived:

(3) $$\begin{array}{c} P\left(X_{i}=o_{j,i}\;|\;X_{P_{i}}=o_{j,P_{i}}\right)=P\left(E_{i}=o_{j,i}-F_{i}\left(o_{j,P_{i}}\right)\;|\;X_{P_{i}}\right)=P\left(E_{i}=o_{j,i}-F_{i}\left(o_{j,P_{i}}\right)\right). \end{array}$$

From Equations (2) and (3), it can be seen that the likelihood over the observational data is equivalent to the likelihood over the noise of the observational data. Let *S* = 〈*G*, *F*〉 denote the causal structure. The likelihood over the noise of the observational data can then be obtained as follows:

(4) $$\begin{array}{c} L\left(S;O\right)=\overset{m}{\underset{j=1}{\sum }}\overset{n}{\underset{i=1}{\sum }}\log\left(P\left(E_{i}=o_{j,i}-F_{i}\left(o_{j,P_{i}}\right)\right.\right). \end{array}$$

Equation (4) shows the converted target function. For datasets with limited sample sizes, the equation must be regularised to avoid the generation of excessive redundant causal edges. By introducing the Bayesian information criterion penalty, the regularised likelihood can be expressed as follows:

(5) $$\begin{array}{c} L_{B}\left(S;O\right)=\overset{n}{\underset{i=1}{\sum }}\left(\overset{m}{\underset{j=1}{\sum }}\log\left(P\left(E_{i}=o_{j,i}-F{\left(o_{j,P_{i}}\right)}_{i}\right)\right.-\frac{d_{i}\log\left(m\right)}{2}\right). \end{array}$$

Equation (5) represents the FCL model, with *d*_*i*_ being the number of coefficients used to estimate *X*_*i*_. By maximising Equation (5), the causal graph structure can be obtained, i.e., max*L*_*B*_(*S*; *O*) = max_*G*_sup_*F*_*L*_*B*_(〈*G*, *F*〉; *O*). This represents the solution process of the FCL-based causal discovery algorithm, which involves two steps: (1) generation of initial causal graphs by fitting and optimising the causal function sup_*F*_*L*_*B*_(〈*G*, *F*〉; *O*); (2) searching for the causal graph with the maximum likelihood max_*G*_*L*_*B*_(〈*G*, *F*〉; *O*) using the hill-climbing algorithm, with the local updating rule for *X*_*i*_ given by the following equation:

(6) $$\begin{array}{c} L_{Bi}^{\prime }\left(S;O\right)=\overset{m}{\underset{j=1}{\sum }}\log\left(P\left(E_{i}=o_{j,i}-F_{i}\left(o_{j,P_{i}}\right)\right)\right)-\frac{d_{i}\log\left(m\right)}{2}. \end{array}$$

The FCL of diabetes risk factors obtained after iteration is denoted as *L*_*B*_^∗^(*S*; *O*). As the termination condition for the hill-climbing algorithm in the search for the causal graph with the maximum target likelihood is *L*_*B*_^∗^(*S*; *O*) > *L*_*B*_(*S*; *O*), where *L*_*B*_(*S*; *O*) is the FCL of the initial causal structure, excessive redundant or erroneous edges are present in the generated diabetes risk factor causal structures. Therefore, an adjustment threshold value is introduced into Equation (5) for correction, resulting in the following corrected model:

(7) $$\begin{array}{c} {\bar{L}}_{B}\left(S;O\right)=\overset{n}{\underset{i=1}{\sum }}\left(\overset{m}{\underset{j=1}{\sum }}\log\left(P\left(E_{i}=o_{j,i}-F_{i}\left(o_{j,P_{i}}\right)\right)\right)-\frac{d_{i}\log\left(m\right)}{2}+\alpha \right). \end{array}$$

Equation (7) represents the modified diabetes risk factor IFCL model, with *α* being the adjustment threshold value. In the hill-climbing algorithm, Equation (6) remains the local updating rule for *X*_*i*_, whereas the termination condition becomes $L_{B}^{*}\left(S;O\right)>{\bar{L}}_{B}\left(S;O\right)$. The likelihood without updated nodes during the iteration process is given by the following equation:

(8) $$\begin{array}{c} L_{Bi}\left(S;O\right)=\overset{m}{\underset{j=1}{\sum }}\log\left(P\left(E_{i}=o_{j,i}-F_{i}\left(o_{j,P_{i}}\right)\right)\right)-\frac{d_{i}\log\left(m\right)}{2}+\alpha . \end{array}$$

The diabetes risk factor FCL of the kth iteration can be expressed as

(9) $$\begin{array}{c} L_{B}^{*}\left(S;O\right)=\overset{n}{\underset{i=1}{\sum }}\left(\overset{m}{\underset{j=1}{\sum }}\log\left(P\left(E_{i}=o_{j,i}-F_{i}\left(o_{j,P_{i}}\right)\right)\right)-\frac{d_{i}\log\left(m\right)}{2}\right)+\alpha_{k} \end{array}$$

where *α*_*k*_ is the total threshold of the *k*th iteration. It can be seen from Equation (7) that the total threshold of the initial IFCL model is *nα*, which can be regarded as the likelihood of each causal node increasing by the threshold *α*, namely, *L*_*Bi*_(*S*; *O*) = ∑_*j*=1_^*m*^log(*P*(*E*_*i*_ = *o*_*j*,*i*_ − *F*_*i*_(*o*_*j*,*P*_*i*__))) − (*d*_*i*_log(*m*)/2) + *α*. After each iteration, the likelihood of updating the node will decrease by *α*, and the total threshold will continue to decrease, namely, *α*_*k*_ < *α*_*l*_, *k* > *l*. Therefore, a causal node with greater likelihood must be searched for in the iteration process to reach the iteration termination condition $L_{B}^{*}\left(S;O\right)>{\bar{L}}_{B}\left(S;O\right)$, which is the fundamental reason why the IFCL-based diabetes risk factor causal discovery algorithm can output a more optimised causal structure.

### 2.2. IFCL-Based Diabetes Risk Factor Causal Discovery Algorithm

**Figure 2:**
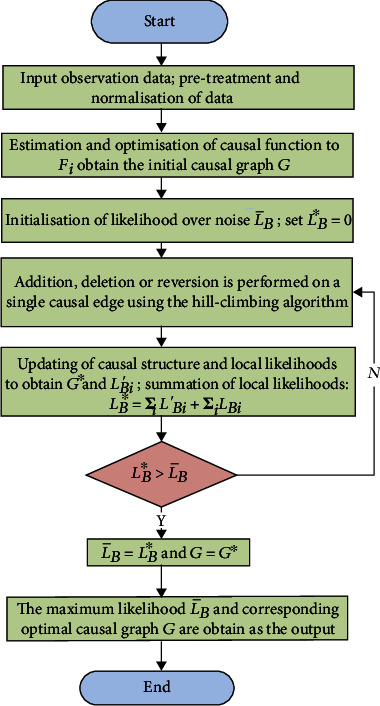
Flowchart of the IFCL-based diabetes risk factor causal discovery algorithm.

Figure 2 shows a flowchart of the IFCL-based diabetes risk factor causal discovery algorithm. The detailed steps of the algorithm are as follows.

**Step 1 .**

The observational data for diabetes risk factors $O=\left\{\vec{o_{1}},\vec{o_{2}},\cdots ,\vec{o_{j}},\cdots ,\vec{o_{m}}\right\}$ are input into the algorithm and subjected to pretreatment and normalisation.

**Step 2 .**

Firstly, the regression method is adopted to estimate the causal function *F*_*i*_ corresponding to the causal edges. Next, the norm of the residual (noise) is calculated by regression. Kernel density estimation is subsequently employed to approximate the noise distribution to obtain the optimised causal function *F*_*i*_, which is then used to generate the initial causal graph *G*.

**Step 3 .**

The likelihood over noise ${\bar{L}}_{B}$ is initialised using Equation (7), and *L*_*B*_^∗^ is set to zero.

**Step 4 .**

The hill-climbing algorithm is used to search for the optimal causal graph. During each iteration, the addition, deletion, or reversion operation is performed on a single causal edge in *G*. The causal function *F*_*i*_ and causal graph are updated, and the updated causal graph is stored in *G*^∗^.

**Step 5 .**

*G* ^∗^ and *G* are compared, and the updating of local likelihoods is performed for nodes with changes using Equation (6) to obtain *L*_*Bi*_′. The updated likelihoods ∑_*i*_*L*_*Bi*_′ and nonupdated likelihoods ∑_*i*_*L*_*Bi*_ are summed to obtain

(10) $$\begin{array}{c} L_{B}^{*}=\underset{i}{\sum }L_{Bi}^{\prime }+\underset{i}{\sum }L_{Bi}. \end{array}$$

**Step 6 .**

*L*_*B*_^∗^ and *L*_*B*_ are compared. If $L_{B}^{*}>{\bar{L}}_{B}$, then ${\bar{L}}_{B}=L_{B}^{*}$ and *G* = *G*^∗^, and the algorithm proceeds to Step 7. Otherwise, Step 4 is executed.

**Step 7 .**

The maximum likelihood ${\bar{L}}_{B}$ and corresponding optimal causal graph *G* are obtained as the output.

## 3. Results and Discussion

### 3.1. Experimental Data and Environment

Diabetes datasets with sample sizes of 768 (denoted as the *M* = 768 dataset) and 2000 (denoted as the *M* = 2000 dataset), which were obtained from the National Institute of Diabetes and Digestive and Kidney Diseases in the U.S.A. and Hospital Frankfurt in Germany, respectively, were downloaded from Kaggle (https://www.kaggle.com/uciml/pima-indians-diabetes-database;https://www.kaggle.com/chirag9073/diabetes-using-deep-learning/data) and used as the experimental data for this study. All subjects in the datasets were at least 21 years old. The datasets consisted of nine variables: number of times pregnant, plasma glucose concentration at 2 h in an oral glucose tolerance test, diastolic blood pressure (mmHg), triceps skin fold thickness (mm), 2 h serum insulin (muU/ml), BMI, diabetes pedigree function, age, and class variable for diabetes diagnosis. In particular, the diabetes pedigree function contains genetic information regarding diabetes history in the family of the subject. Except for the class variable, all other variables were subjected to causality analysis in this study. To maximise the retention of information, mean imputation was adopted to replace the missing values in the datasets. *Z*-score standardisation was performed on the raw data, and abnormal values were replaced by mean values.

The simulation experiment was carried out in the RStudio environment, and the program was written in R language. The computer used had an Intel (R) Core (TM) i7-6500U CPU with main frequency 2.50 GHz and 8 GB of RAM.

### 3.2. Experimental Results

#### 3.2.1. Scatter Plots and Correlation Coefficients of Variable Pairs

**Figure 3:**
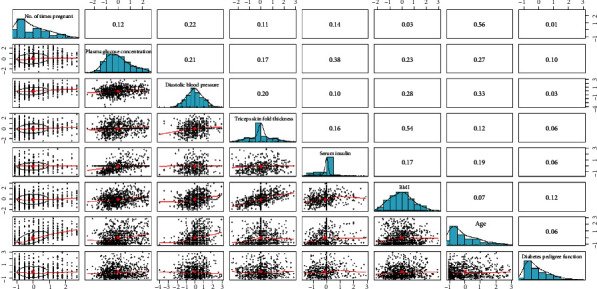
Scatter plots, bar charts, and correlation coefficients for the *M* = 768 dataset.

**Figure 4:**
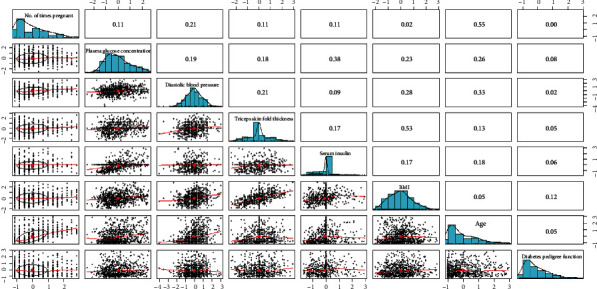
Scatter plots, bar charts, and correlation coefficients for the *M* = 2000 dataset.

To understand their correlation and provide a basis for subsequent experiments to analyse their causality, the scatter plots and correlation coefficients of variable pairs among the eight variables were generated for the *M* = 768 dataset (Figure 3) and *M* = 2000 dataset (Figure 4).

Figures 3 and 4 show scatter plots of the variable pairs in the bottom left corner, bar charts for each variable on the diagonal line from top left to bottom right, and correlation coefficients of the variable pairs in the top right corner. Figures 3 and 4 both show the scatter plots and correlation coefficients of 28 variable pairs. There are seven variable pairs with correlation coefficients less than 0.1 in Figure 3, while there are eight pairs of such cases in Figure 4.

In general, if the correlation coefficient of two variables is between 0 and 0.1, the relationship between the variables can be considered nonlinear. Therefore, variable pairs with correlation coefficients < 0.1 were discarded. Tables 1 and 2 show the variable pairs with correlation coefficients ≥ 0.1 and the corresponding *P* values. All *P* values are less than 0.01, which indicates the existence of significant linear relationships in the variable pairs.

#### 3.2.2. Results of FCL-Based Causal Discovery

To better demonstrate and analyse the causal structure of diabetes risk factors, we set no. of times pregnant, plasma glucose concentration, diastolic blood pressure, triceps skin fold thickness, insulin, BMI, age, and diabetes pedigree function to the variables *X*_1_, *X*_2_, *X*_3_, *X*_4_, *X*_5_, *X*_6_, *X*_7_, and *X*_8_, respectively.

**Figure 5:**
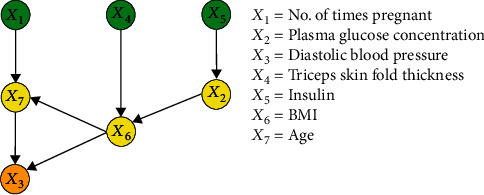
Causal structure for the *M* = 768 dataset (structure 1).

**Figure 6:**
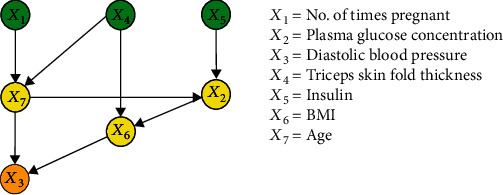
Causal structure for the *M* = 2000 dataset (structure 2).

To investigate the presence or absence of causality among the eight variables, a causal discovery experiment was performed with the *M* = 768 and *M* = 2000 datasets using an FCL-based causal discovery algorithm reported previously [33]. Figures 5 and 6 depict the resultant causal structures (named structures 1 and 2) and show 7 and 8 pairs of causal relationships, respectively. In Figures 5 and 6, green nodes represent the ancestor nodes, which have only child nodes; yellow nodes represent the intermediate nodes, which have both parent and child nodes; and orange nodes represent the child nodes, which have only parent nodes.  Table 3 shows the maximum likelihoods for both structures.

- Similarities between structures 1 and 2: both structures exhibit six identical causal relationships: *X*_1_ → *X*_7_, *X*_7_ → *X*_3_, *X*_4_ → *X*_6_, *X*_5_ → *X*_2_, *X*_2_ → *X*_6_, and *X*_6_ → *X*_3_, with *X*_1_ → *X*_7_ indicating that the number of times pregnant causes changes in age, *X*_7_ → *X*_3_ indicating that age causes changes in diastolic blood pressure, *X*_4_ → *X*_6_ indicating that triceps skin fold thickness causes changes in BMI, *X*_5_ → *X*_2_ indicating that insulin causes changes in plasma glucose concentration, *X*_2_ → *X*_6_ indicating that plasma glucose concentration causes changes in BMI, and *X*_6_ → *X*_3_ indicating that BMI causes changes in diastolic blood pressure. There was an absence of causal relationships between diabetes pedigree function and all other variables in both structures
- Differences between structures 1 and 2: structure 1 exhibits the causal relationship *X*_6_ → *X*_7_, whereas structure 2 shows the causal relationships *X*_7_ → *X*_2_ and *X*_4_ → *X*_7_, with *X*_6_ → *X*_7_ indicating that BMI causes changes in age, *X*_7_ → *X*_2_ indicating that age causes changes in plasma glucose concentration, and *X*_4_ → *X*_7_ indicating that triceps skin fold thickness causes changes in age

Figure 3 shows that the correlation coefficient between BMI and age is 0.07, and the corresponding *P* value is 0.072. Therefore, the absence of a linear relationship between BMI and age can be deduced. Obviously, the causal function obtained by the regression method fails the significance test and has no statistical significance. On this basis, *X*_6_ → *X*_7_ can be regarded as an erroneous causal relationship. In Figure 6, the erroneous causal edge *X*_6_ → *X*_7_ was eliminated when causal discovery was performed with the *M* = 2000 dataset, but two additional causal edges *X*_4_ → *X*_7_ and *X*_7_ → *X*_2_ were discovered. As shown in Table 3, the maximum likelihood of structure 2 is higher than that of structure 1, which suggests that sample size influences the results of causal discovery. Although a larger sample size favours the elimination of erroneous causal edges and discovery of previously nonexistent causal edges, the increase in the number of discovered causal edges may also lead to an increase in the number of redundant edges. Figures 5 and 6 demonstrate that the causal structures were complex with significant numbers of redundant or erroneous edges, which necessitates the development of new causal discovery algorithms.

#### 3.2.3. Results of IFCL-Based Causal Discovery

The purpose of this experiment was to compare the performance of the proposed method with the FCL-based causal discovery method and explore the optimal causal structure of diabetes risk factors. When the IFCL-based algorithm was adopted for causal discovery in the *M* = 768 and *M* = 2000 datasets, it was found that the results of causal discovery were closely associated with the adjustment threshold value. In the experiment, *α* values of 0.05 ≤ *α* ≤ 0.18 in intervals of 0.01 were adopted, whereas *α* values < 0.05 were not used owing to the generation of excessive redundant causal edges.

**Figure 7:**
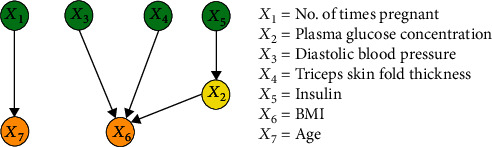
Causal structure for the *M* = 768 dataset (*α* = 0.05–0.06) (structure 3).

**Figure 8:**
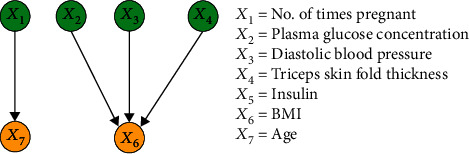
Causal structure for the *M* = 768 dataset (*α* = 0.07–0.14) (structure 4).

**Figure 9:**
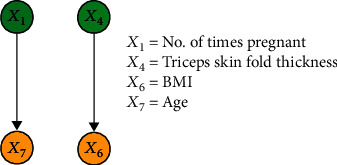
Causal structure for the *M* = 768 dataset (*α* = 0.15) (structure 5).

**Figure 10:**
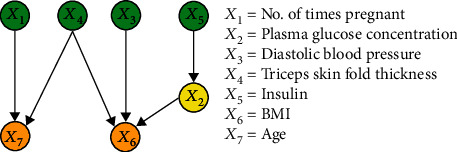
Causal structure for the *M* = 2000 dataset (*α* = 0.05–0.06) (structure 6).

**Figure 11:**
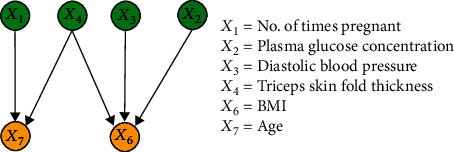
Causal structure for the *M* = 2000 dataset (*α* = 0.07–0.15) (structure 7).

**Figure 12:**
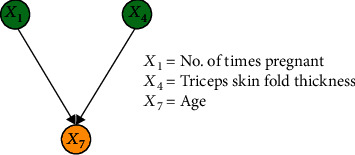
Causal structure for the *M* = 2000 dataset (*α* = 0.18) (structure 8).

- Causal structures for the *M* = 768 dataset: Figure 7 shows the generated causal structure with five pairs of causal relationships (*X*_1_ → *X*_7_, *X*_3_ → *X*_6_, *X*_4_ → *X*_6_, *X*_5_ → *X*_2_, and *X*_2_ → *X*_6_) when *α* = 0.05–0.06 (structure 3). In structure 3, *X*_1_, *X*_3_, *X*_4_, and *X*_5_ are the ancestor nodes, *X*_2_ is the intermediate node, and *X*_6_ and *X*_7_ are the child nodes. Compared with structure 1, *X*_6_ → *X*_7_ (an erroneous edge) and *X*_7_ → *X*_3_ are absent, and *X*_6_ → *X*_3_ is reversed to form the *X*_3_ → *X*_6_ relationship in structure 3.  Figure 8 shows the generated causal structure with four pairs of causal relationships (*X*_1_ → *X*_7_, *X*_2_ → *X*_6_, *X*_3_ → *X*_6_, and *X*_4_ → *X*_6_) when *α* = 0.07–0.14 (structure 4). In structure 4, *X*_1_, *X*_2_, *X*_3_, and *X*_4_ are the ancestor nodes, *X*_7_ and *X*_6_ are the child nodes, and there is no intermediate node. Compared with structure 3, the causal edge *X*_5_ → *X*_2_ is absent in structure 4.  Figure 9 shows the generated causal structure when *α* = 0.15 (structure 5), which merely consists of two causal edges, *X*_1_ → *X*_7_ and *X*_4_ → *X*_6_. In structure 5, there are only the ancestor nodes (*X*_1_ and *X*_4_) and child nodes (*X*_7_ and *X*_6_). Further simplification did not occur in the causal structure when *α* was increased beyond 0.15
- Causal structures for the *M* = 2000 dataset: Figure 10 shows the generated causal structure with six pairs of causal relationships (*X*_1_ → *X*_7_, *X*_4_ → *X*_7_, *X*_3_ → *X*_6_, *X*_4_ → *X*_6_, *X*_5_ → *X*_2_, and *X*_2_ → *X*_6_) when *α* = 0.05–0.06 (structure 6). In structure 6, *X*_1_, *X*_3_, *X*_4_, and *X*_5_ are the ancestor nodes, *X*_2_ is the intermediate node, and *X*_6_ and *X*_7_ are child nodes. Compared with structure 2, the causal edges *X*_7_ → *X*_3_ and *X*_7_ → *X*_2_ are absent, and *X*_6_ → *X*_3_ is reversed to form the *X*_3_ → *X*_6_ relationship in structure 6.  Figure 11 shows the generated causal structure with five pairs of causal relationships (*X*_1_ → *X*_7_, *X*_4_ → *X*_7_, *X*_4_ → *X*_6_, *X*_3_ → *X*_6_, and *X*_2_ → *X*_6_) when *α* = 0.07–0.15 (structure 7). In structure 7, *X*_1_, *X*_2_, *X*_3_, and *X*_4_ are the ancestor nodes, *X*_7_ and *X*_6_ are the child nodes, and there is no intermediate node. Compared with structure 6, *X*_5_ → *X*_2_ is absent from structure 7. When *α* = 0.16–0.17, the algorithm could not find an optimal causal structure.  Figure 12 shows the generated causal structure when *α* ≥ 0.18 (structure 8), which merely consists of two causal edges, *X*_1_ → *X*_7_ and *X*_4_ → *X*_7_. In structure 8, there are only the ancestor nodes (*X*_1_ and *X*_4_) and a child node (*X*_7_). Additional changes did not occur in the causal structure when *α* was increased further

Table 4 shows the maximum likelihoods for structures 3–8. It can be seen that the maximum likelihood increased with increasing sample size.

The results presented above indicate that a larger sample size leads to a reduction in the number of erroneous causal relationships and the discovery of other potential causal relationships. During the causal discovery process, *α* must be incorporated to reduce the number of redundant and erroneous edges. When *α* was increased, the causal structures generated using the improved algorithm proposed in this study became increasingly simplified. In particular, when *α* was set to 0.05 or 0.06, causal structures with the fewest redundant edges and maximum information retention were obtained. Therefore, it can be deduced that the optimal adjustment threshold values for the discovery of causal relationships among the diabetes risk factors were 0.05 and 0.06.

## 4. Analysis and Discussion

As shown in Figures 7 and 10, a total of six causal relationships (*X*_5_ → *X*_2_, *X*_2_ → *X*_6_, *X*_4_ → *X*_6_, *X*_3_ → *X*_6_, *X*_1_ → *X*_7_, and *X*_4_ → *X*_7_), which are discussed in detail below, existed among the various diabetes risk factors.

1. *X*_5_ → *X*_2_, *X*_2_ → *X*_6_: these causal relationships are well known among the general public. Insulin is the only hormone that lowers blood glucose levels in the human body. If insulin resistance occurs, abnormalities will arise in glucose uptake in the body, which will lead to increased plasma glucose concentration and result in a higher likelihood of diabetes onset. Additionally, *X*_5_ → *X*_2_ and *X*_2_ → *X*_6_ can be combined to form the causal relationship *X*_5_ → *X*_2_ → *X*_6_. In a typical human body with normal insulin secretion, blood glucose metabolism will be at a standard level, which will lead to the maintenance of normal BMI. In contrast, in diabetic patients with insulin resistance, blood glucose cannot be effectively absorbed and utilised, which leads to decreased body weight and lower BMI. Therefore, the causal relationship *X*_5_ → *X*_2_ → *X*_6_ also holds true
2. *X*_4_ → *X*_6_: the triceps skin fold thickness reflects body fat content, with a greater thickness indicating a higher body fat percentage and body weight, which leads to an increase in BMI and risk of diabetes onset. When diabetes causes emaciation in patients, triceps skin fold thickness and body weight are reduced, causing a decrease in BMI. Therefore, the causal relationship *X*_4_ → *X*_6_ still holds true
3. *X*_3_ → *X*_6_: when causal discovery was performed in accordance with a previously reported method [33], the discovered relationship between factors 3 and 6 was *X*_6_ → *X*_3_ (as shown in Figures 5 and 6), i.e., BMI influenced diastolic blood pressure. As people with higher body fat contents have higher BMIs and increased tendencies to develop hypertension, such a causal relationship is consistent with common medical knowledge and indicates that BMI is a trigger for hypertension. However, when causal discovery was performed using the modified method developed in this study, the reverse relationship (*X*_3_ → *X*_6_) was discovered (as shown in Figures 7 and 10). This finding suggests the possible existence of a certain casual factor that changed under the influence of BMI and consequently influenced the risk of diabetes onset. Notably, certain diabetic patients suffer from concomitant hypertension and emaciation. Medical professionals generally believe that emaciation is caused by diabetes, but it may also be jointly influenced by diabetes and hypertension, resulting in changes in BMI. Therefore, *X*_3_ → *X*_6_ may be a little-known relationship that exists in reality
4. *X*_1_ → *X*_7_: this causal relationship indicates that the number of times pregnant causes changes in age. In a previous study [35], it was reported that an increased number of pregnancies was associated with higher physiological age, i.e., cellular ageing may be accelerated, which in turn causes a higher probability of developing certain diseases. Therefore, the causal mechanism underlying *X*_1_ → *X*_7_ may be as follows: an increased number of times pregnant causes accelerated ageing of pancreatic *β* cells, which leads to a higher tendency to develop insulin resistance and an increased diabetes risk
5. *X*_4_ → *X*_7_: this causal relationship indicates that the triceps skin fold thickness causes changes in age. As the triceps skin fold thickness reflects the nutritional status of an individual, the underlying causal mechanism for *X*_4_ → *X*_7_ may be as follows: a triceps skin fold thickness that is less than the standard value indicates malnutrition, which affects physiological age and causes pancreatic *β* cell ageing, thereby causing insulin resistance and an increased diabetes risk. An excessively large triceps skin fold thickness indicates obesity, which signifies the presence of an excessive amount of glucose in the body. Consequently, the pancreatic *β* cells become overworked for long periods, which increases the tendency for ageing and functional damage in the pancreas, resulting in an increased risk of diabetes

In short, among the causal relationships identified through the IFCL-based causal discovery method proposed in this study, *X*_5_ → *X*_2_ → *X*_6_ and *X*_4_ → *X*_6_ are confirmed relationships, whereas *X*_3_ → *X*_6_, *X*_1_ → *X*_7_, and *X*_4_ → *X*_7_ require further validation. These results suggest that the improved algorithm possesses huge potential for the discovery of causal relationships among diabetes risk factors and may be beneficial for further elucidation of causality among diabetes risk factors.

## 5. Conclusion

In the present study, we proposed an IFCL-based diabetes risk factor causal discovery algorithm that effectively resolves the issue of excessive redundant and erroneous edges in the causal structures generated by the FCL-based algorithm. Our experimental results demonstrate the efficacy of the proposed algorithm and provide a scientific basis for uncovering causal relationships among various diabetes risk factors. The next step in our research efforts will be the exploration of causality among the biochemical markers of diabetes and physiological indicators of body composition, with the objective of elucidating the causal relationships between the pathological and physiological factors of diabetes and enhancing diabetes prevention and treatment efforts.

## Figures and Tables

**Table 1:** Correlation coefficients and *P* values of variable pairs for the *M* = 768 dataset.

Variable pair	Correlation coefficient	*P* value
No. of times pregnant and age	0.56	0
No. of times pregnant and diastolic blood pressure	0.21	0
No. of times pregnant and insulin	0.14	0
No. of times pregnant and triceps skin fold thickness	0.11	0.003
No. of times pregnant and plasma glucose concentration	0.11	0.001
Plasma glucose concentration and insulin	0.38	0
Plasma glucose concentration and age	0.27	0
Plasma glucose concentration and BMI	0.23	0
Plasma glucose concentration and diastolic blood pressure	0.21	0
Plasma glucose concentration and triceps skin fold thickness	0.17	0
Plasma glucose concentration and diabetes pedigree function	0.10	0.005
Diastolic blood pressure and age	0.33	0
Diastolic blood pressure and BMI	0.28	0
Diastolic blood pressure and triceps skin fold thickness	0.20	0
Diastolic blood pressure and insulin	0.10	0.005
Triceps skin fold thickness and BMI	0.54	0
Triceps skin fold thickness and insulin	0.16	0
Triceps skin fold thickness and age	0.12	0.001
Insulin and age	0.19	0
Insulin and BMI	0.17	0
BMI and diabetes pedigree function	0.12	0.001

**Table 2:** Correlation coefficients and *P* values of variable pairs for the *M* = 2000 dataset.

Variable pair	Correlation coefficient	*P* value
No. of times pregnant and age	0.55	0
No. of times pregnant and diastolic blood pressure	0.21	0
No. of times pregnant and insulin	0.11	0
No. of times pregnant and triceps skin fold thickness	0.11	0
No. of times pregnant and plasma glucose concentration	0.11	0
Plasma glucose concentration and insulin	0.38	0
Plasma glucose concentration and age	0.26	0
Plasma glucose concentration and BMI	0.23	0
Plasma glucose concentration and diastolic blood pressure	0.19	0
Plasma glucose concentration and triceps skin fold thickness	0.18	0
Diastolic blood pressure and age	0.33	0
Diastolic blood pressure and BMI	0.28	0
Diastolic blood pressure and triceps skin fold thickness	0.21	0
Triceps skin fold thickness and BMI	0.53	0
Triceps skin fold thickness and insulin	0.17	0
Triceps skin fold thickness and age	0.13	0
Insulin and age	0.18	0
Insulin and BMI	0.17	0
BMI and diabetes pedigree function	0.12	0

**Table 3:** Maximum likelihoods of causal structures 1 and 2.

Causal structure	Maximum likelihood
1	-8.34
2	-8.17

**Table 4:** Maximum likelihoods for causal structures 3–8.

Dataset	Causal structure	Maximum likelihood
*M* = 768	3 (*α* = 0.05–0.06)	-8.18, -8.13
	4 (*α* = 0.07–0.14)	-8.09, -8.03, -7.97, -7.91, -7.86, -7.79, -7.73, -7.67
	5 (*α* = 0.15)	-7.63
*M* = 2000	6 (*α* = 0.05–0.06)	-8.01, -7.96
	7 (*α* = 0.07–0.15)	-7.92, -7.86, -7.80, -7.74, -7.68, -7.62, -7.56, -7.50, -7.44
	8 (*α* = 0.18)	-7.26

## Data Availability

The data used to support the findings of this study are included within the supplementary information files.

## Supplementary Materials

Supplementary Materials See Datasets S1–S2, Source Code S3, and Editorial Certificate S4 in the Supplementary Material for simulation experiments.
