# Supplementary material for: Improved Functional Causal Likelihood-Based Causal Discovery Method for Diabetes Risk Factors
Source: Comput Math Methods Med. 2021 May 14;2021:5552085. doi: 10.1155/2021/5552085 (PMC8143882; doi:10.1155/2021/5552085)
Supplement: Supplementary Materials — See Datasets S1–S2, Source Code S3, and Editorial Certificate S4 in the Supplementary Material for simulation experiments. [file 5552085.f1.zip › S1.pdf]

PregnanciGlucose		BloodPresSkinThickInsulin			BMI	DiabetesFAge		Outcome
0	102	52	29.15	155.55	25.1	0.078	21	0
2	108	62	32	56	25.2	0.128	21	0
3	111	62	29.15	155.55	22.6	0.142	21	0
0	137	68	14	148	24.8	0.143	21	0
1	97	70	15	155.55	18.2	0.147	21	0
4	94	65	22	155.55	24.7	0.148	21	0
2	112	75	32	155.55	35.7	0.148	21	0
2	108	64	29.15	155.55	30.8	0.158	21	0
1	89	66	23	94	28.1	0.167	21	0
1	105	58	29.15	155.55	24.3	0.187	21	0
1	91	64	24	155.55	29.2	0.192	21	0
1	116	70	28	155.55	27.4	0.204	21	0
0	125	68	29.15	155.55	24.7	0.206	21	0
0	139	62	17	210	22.1	0.207	21	0
4	99	76	15	51	23.2	0.223	21	0
1	73	50	10	155.55	23	0.248	21	0
0	101	64	17	155.55	21	0.252	21	0
1	143	74	22	61	26.2	0.256	21	0
1	80	55	29.15	155.55	19.1	0.258	21	0
0	125	96	29.15	155.55	22.5	0.262	21	0
2	130	96	29.15	155.55	22.6	0.268	21	0
1	96	64	27	87	33.2	0.289	21	0
1	114	66	36	200	38.1	0.289	21	0
1	97	64	19	82	18.2	0.299	21	0
1	121.69	74	20	23	27.7	0.299	21	0
2	84	72.41	29.15	155.55	32.46	0.304	21	0
2	117	90	19	71	25.2	0.313	21	0
0	94	70	27	115	43.5	0.347	21	0
0	134	58	20	291	26.4	0.352	21	0
2	100	64	23	155.55	29.7	0.368	21	0
0	151	90	46	155.55	42.1	0.371	21	1
1	131	64	14	415	23.7	0.389	21	0
0	132	78	29.15	155.55	32.4	0.393	21	0
1	99	72	30	18	38.6	0.412	21	0
2	115	64	22	155.55	30.8	0.421	21	0
1	71	78	50	45	33.2	0.422	21	0
0	78	88	29	40	36.9	0.434	21	0
3	87	60	18	155.55	21.8	0.444	21	0
0	124	56	13	105	21.8	0.452	21	0
2	99	60	17	160	36.6	0.453	21	0
0	102	64	46	78	40.6	0.496	21	0
1	124	60	32	155.55	35.8	0.514	21	0
0	126	86	27	120	27.4	0.515	21	0
0	138	60	35	167	34.6	0.534	21	1
1	139	62	41	480	40.7	0.536	21	0
1	113	64	35	155.55	33.6	0.543	21	1
0	84	64	22	66	35.8	0.545	21	0
1	99	58	10	155.55	25.4	0.551	21	0
1	89	24	19	25	27.8	0.559	21	0
3	90	78	29.15	155.55	42.7	0.559	21	0

2	94	68	18	76	26	0.561	21	0
0	102	75	23	155.55	32.46	0.572	21	0
0	100	70	26	50	30.8	0.597	21	0
2	99	52	15	94	24.6	0.637	21	0
1	130	60	23	170	28.6	0.692	21	0
2	118	80	29.15	155.55	42.9	0.693	21	1
2	142	82	18	64	24.7	0.761	21	0
1	126	56	29	152	28.7	0.801	21	0
0	113	80	16	155.55	31	0.874	21	0
1	109	60	8	182	25.4	0.947	21	0
2	84	50	23	76	30.4	0.968	21	0
0	177	60	29	478	34.6	1.072	21	1
0	118	64	23	89	32.46	1.731	21	0
2	90	70	17	155.55	27.3	0.085	22	0
1	87	78	27	32	34.6	0.101	22	0
2	74	72.41	29.15	155.55	32.46	0.102	22	0
1	128	82	17	183	27.5	0.115	22	0
3	113	44	13	155.55	22.4	0.14	22	0
1	121.69	48	20	155.55	24.7	0.14	22	0
2	92	52	29.15	155.55	30.1	0.141	22	0
1	106	70	28	135	34.2	0.142	22	0
2	101	58	35	90	21.8	0.155	22	0
0	137	70	38	155.55	33.2	0.17	22	0
0	105	64	41	142	41.5	0.173	22	0
3	80	72.41	29.15	155.55	32.46	0.174	22	0
2	127	46	21	335	34.4	0.176	22	0
1	151	60	29.15	155.55	26.1	0.179	22	0
0	131	66	40	155.55	34.3	0.196	22	1
2	98	60	17	120	34.7	0.198	22	0
1	109	58	18	116	28.5	0.219	22	0
2	88	74	19	53	29	0.229	22	0
0	105	68	22	155.55	20	0.236	22	0
0	101	65	28	155.55	24.6	0.237	22	0
0	99	72.41	29.15	155.55	25	0.253	22	0
3	148	66	25	155.55	32.5	0.256	22	0
3	173	84	33	474	35.7	0.258	22	1
8	120	86	29.15	155.55	28.4	0.259	22	1
0	119	66	27	155.55	38.8	0.259	22	0
1	95	60	18	58	23.9	0.26	22	0
3	88	58	11	54	24.8	0.267	22	0
0	74	52	10	36	27.8	0.269	22	0
0	98	82	15	84	25.2	0.299	22	0
3	81	86	16	66	27.5	0.306	22	0
3	130	64	29.15	155.55	23.1	0.314	22	0
2	108	52	26	63	32.5	0.318	22	0
1	71	48	18	76	20.4	0.323	22	0
2	175	88	29.15	155.55	22.9	0.326	22	0
2	56	56	28	45	24.2	0.332	22	0
0	95	64	39	105	44.6	0.366	22	0
1	121.69	68	35	155.55	32	0.389	22	0
1	79	75	30	155.55	32	0.396	22	0

3	139	54	29.15	155.55	25.6	0.402	22	1
1	93	56	11	155.55	22.5	0.417	22	0
2	106	56	27	165	29	0.426	22	0
3	111	58	31	44	29.5	0.43	22	0
0	141	84	26	155.55	32.4	0.433	22	0
4	97	60	23	155.55	28.2	0.443	22	0
4	91	70	32	88	33.1	0.446	22	0
0	179	50	36	159	37.8	0.455	22	1
1	130	70	13	105	25.9	0.472	22	0
1	97	66	15	140	23.2	0.487	22	0
1	103	80	11	82	19.4	0.491	22	0
0	117	66	31	188	30.8	0.493	22	0
2	83	66	23	50	32.2	0.497	22	0
1	87	60	37	75	37.2	0.509	22	0
0	104	64	37	64	33.6	0.51	22	1
2	91	62	29.15	155.55	27.3	0.525	22	0
1	80	74	11	60	30	0.527	22	0
0	93	60	25	92	28.7	0.532	22	0
1	79	80	25	37	25.4	0.583	22	0
2	71	70	27	155.55	28	0.586	22	0
0	106	70	37	148	39.4	0.605	22	0
4	142	86	29.15	155.55	44	0.645	22	1
1	139	46	19	83	28.7	0.654	22	0
0	188	82	14	185	32	0.682	22	1
2	122	60	18	106	29.8	0.717	22	0
2	95	54	14	88	26.1	0.748	22	0
2	88	58	26	16	28.4	0.766	22	0
3	123	100	35	240	57.3	0.88	22	0
2	108	62	10	278	25.3	0.881	22	0
1	143	84	23	310	42.4	1.076	22	0
1	97	68	21	155.55	27.2	1.095	22	0
4	136	70	29.15	155.55	31.2	1.182	22	1
1	181	78	42	293	40	1.258	22	1
1	199	76	43	155.55	42.9	1.394	22	1
2	99	72.41	29.15	155.55	22.2	0.108	23	0
2	102	86	36	120	45.5	0.127	23	1
0	107	60	25	155.55	26.4	0.133	23	0
1	111	62	13	182	24	0.138	23	0
1	111	86	19	155.55	30.1	0.143	23	0
3	116	72.41	29.15	155.55	23.5	0.187	23	0
1	89	76	34	37	31.2	0.192	23	0
4	129	86	20	270	35.1	0.231	23	0
0	84	82	31	125	38.2	0.233	23	0
1	91	54	25	100	25.2	0.234	23	0
4	154	62	31	284	32.8	0.237	23	0
2	68	62	13	15	20.1	0.257	23	0
1	118	58	36	94	33.3	0.261	23	0
0	113	76	29.15	155.55	33.3	0.278	23	1
3	74	68	28	45	29.7	0.293	23	0
4	132	72.41	29.15	155.55	32.9	0.302	23	1
1	93	70	31	155.55	30.4	0.315	23	0

2	111	60	29.15	155.55	26.2	0.343	23	0
6	93	50	30	64	28.7	0.356	23	0
2	107	74	30	100	33.6	0.404	23	0
1	82	64	13	95	21.2	0.415	23	0
1	88	62	24	44	29.9	0.422	23	0
0	165	90	33	680	52.3	0.427	23	0
0	104	64	23	116	27.8	0.454	23	0
2	134	70	29.15	155.55	28.9	0.542	23	1
2	101	58	17	265	24.2	0.614	23	0
2	94	76	18	66	31.6	0.649	23	0
2	93	64	32	160	38	0.674	23	1
3	107	62	13	48	22.9	0.678	23	1
1	79	60	42	48	43.5	0.678	23	0
0	179	90	27	155.55	44.1	0.686	23	1
1	153	82	42	485	40.6	0.687	23	0
0	119	64	18	92	34.9	0.725	23	0
0	127	80	37	210	36.3	0.804	23	0
1	140	74	26	180	24.1	0.828	23	0
1	109	56	21	135	25.2	0.833	23	0
2	121	70	32	95	39.1	0.886	23	0
1	143	86	30	330	30.1	0.892	23	0
0	117	80	31	53	45.2	0.089	24	0
3	116	74	15	105	26.3	0.107	24	0
1	157	72	21	168	25.6	0.123	24	0
4	114	64	29.15	155.55	28.9	0.126	24	0
2	92	62	28	155.55	31.6	0.13	24	0
0	119	72.41	29.15	155.55	32.4	0.141	24	1
3	125	58	29.15	155.55	31.6	0.151	24	0
3	99	54	19	86	25.6	0.154	24	0
1	107	68	19	155.55	26.5	0.165	24	0
2	112	78	50	140	39.4	0.175	24	0
3	171	72	33	135	33.3	0.199	24	1
1	119	54	13	50	22.3	0.205	24	0
1	112	80	45	132	34.8	0.217	24	0
4	117	64	27	120	33.2	0.23	24	0
0	102	78	40	90	34.5	0.238	24	0
0	95	85	25	36	37.4	0.247	24	1
2	90	80	14	55	24.4	0.249	24	0
6	111	64	39	155.55	34.2	0.26	24	0
1	81	72	18	40	26.6	0.283	24	0
3	106	54	21	158	30.9	0.292	24	0
3	158	64	13	387	31.2	0.295	24	0
4	128	70	29.15	155.55	34.3	0.303	24	0
7	105	72.41	29.15	155.55	32.46	0.305	24	0
2	112	66	22	155.55	25	0.307	24	0
5	86	68	28	71	30.2	0.364	24	0
0	135	68	42	250	42.3	0.365	24	1
0	147	85	54	155.55	42.8	0.375	24	0
1	136	74	50	204	37.4	0.399	24	0
1	108	88	19	155.55	27.1	0.4	24	0
1	87	68	34	77	37.6	0.401	24	0

1	108	60	46	178	35.5	0.415	24	0
0	140	65	26	130	42.6	0.431	24	1
2	100	54	28	105	37.8	0.498	24	0
0	126	84	29	215	30.7	0.52	24	0
1	90	62	12	43	27.2	0.58	24	0
4	95	70	32	155.55	32.1	0.612	24	0
1	128	48	45	194	40.5	0.613	24	1
2	83	65	28	66	36.8	0.629	24	0
2	174	88	37	120	44.5	0.646	24	1
3	162	52	38	155.55	37.2	0.652	24	1
1	193	50	16	375	25.9	0.655	24	0
0	107	76	29.15	155.55	45.3	0.686	24	0
2	141	58	34	128	25.4	0.699	24	0
1	107	72	30	82	30.8	0.821	24	0
2	128	64	42	155.55	40	1.101	24	0
1	77	56	30	56	33.3	1.251	24	0
2	114	68	22	155.55	28.7	0.092	25	0
3	121	52	29.15	155.55	36	0.127	25	1
4	84	90	23	56	39.5	0.159	25	0
2	87	58	16	52	32.7	0.166	25	0
2	68	70	32	66	25	0.187	25	0
2	90	60	29.15	155.55	23.5	0.191	25	0
3	112	74	30	155.55	31.6	0.197	25	1
1	125	70	24	110	24.3	0.221	25	0
3	108	62	24	155.55	26	0.223	25	0
2	105	58	40	94	34.9	0.225	25	0
0	86	68	32	155.55	35.8	0.238	25	0
2	155	52	27	540	38.7	0.24	25	1
3	193	70	31	155.55	34.9	0.241	25	1
0	94	72.41	29.15	155.55	32.46	0.256	25	0
0	93	60	29.15	155.55	35.3	0.263	25	0
1	119	44	47	63	35.5	0.28	25	0
2	81	60	22	155.55	27.7	0.29	25	0
1	95	66	13	38	19.6	0.334	25	0
0	101	62	29.15	155.55	21.9	0.336	25	0
3	83	58	31	18	34.3	0.336	25	0
0	73	72.41	29.15	155.55	21.1	0.342	25	0
5	139	80	35	160	31.6	0.361	25	1
0	91	68	32	210	39.9	0.381	25	0
3	82	70	29.15	155.55	21.1	0.389	25	0
4	76	62	29.15	155.55	34	0.391	25	0
2	144	58	33	135	31.6	0.422	25	1
1	92	62	25	41	19.5	0.482	25	0
1	116	78	29	180	36.1	0.496	25	0
5	109	62	41	129	35.8	0.514	25	1
1	112	72	30	176	34.4	0.528	25	0
2	81	72	15	76	30.1	0.547	25	0
3	84	68	30	106	31.9	0.591	25	0
2	129	74	26	205	33.2	0.591	25	0
0	97	64	36	100	36.8	0.6	25	0
3	113	50	10	85	29.5	0.626	25	0

5	78	48	29.15	155.55	33.7	0.654	25	0
2	100	70	52	57	40.5	0.677	25	0
0	107	62	30	74	36.6	0.757	25	1
0	162	76	56	100	53.2	0.759	25	1
2	87	72.41	23	155.55	28.9	0.773	25	0
0	138	72.41	29.15	155.55	36.3	0.933	25	1
1	90	62	18	59	25.1	1.268	25	0
0	128	68	19	180	30.5	1.391	25	1
2	127	58	24	275	27.7	1.6	25	0
2	82	52	22	115	28.5	1.699	25	0
0	180	66	39	155.55	42	1.893	25	1
3	173	82	48	465	38.4	2.137	25	1
0	180	78	63	14	59.4	2.42	25	1
6	114	72.41	29.15	155.55	32.46	0.189	26	0
1	106	76	29.15	155.55	37.5	0.197	26	0
0	101	76	29.15	155.55	35.7	0.198	26	0
0	181	88	44	510	43.3	0.222	26	1
3	78	50	32	88	31	0.248	26	1
0	165	76	43	255	47.9	0.259	26	0
0	131	72.41	29.15	155.55	43.2	0.27	26	1
3	180	64	25	70	34	0.271	26	0
3	99	62	19	74	21.8	0.279	26	0
0	135	94	46	145	40.6	0.284	26	0
0	120	74	18	63	30.5	0.285	26	0
3	124	80	33	130	33.2	0.305	26	0
2	112	68	22	94	34.1	0.315	26	0
0	129	110	46	130	67.1	0.319	26	1
2	100	68	25	71	38.5	0.324	26	0
0	95	80	45	92	36.5	0.33	26	0
4	96	56	17	49	20.8	0.34	26	0
0	162	76	36	155.55	49.6	0.364	26	1
3	102	44	20	94	30.8	0.4	26	0
1	109	38	18	120	23.1	0.407	26	0
5	139	64	35	140	28.6	0.411	26	0
1	71	62	29.15	155.55	21.8	0.416	26	0
4	171	72	29.15	155.55	43.6	0.479	26	1
2	122	76	27	200	35.9	0.483	26	0
1	88	30	42	99	55	0.496	26	1
1	119	88	41	170	45.3	0.507	26	0
2	123	48	32	165	42.1	0.52	26	0
1	101	50	15	36	24.2	0.526	26	0
2	96	68	13	49	21.1	0.647	26	0
1	100	66	15	56	23.6	0.666	26	0
6	105	80	28	155.55	32.5	0.878	26	0
4	118	70	29.15	155.55	44.5	0.904	26	0
4	109	64	44	99	34.8	0.905	26	1
4	110	76	20	100	28.4	0.118	27	0
0	114	80	34	285	44.2	0.167	27	0
4	146	85	27	100	28.9	0.189	27	0
6	109	60	27	155.55	25	0.206	27	0
1	96	122	29.15	155.55	22.4	0.207	27	0

3	106	72	29.15	155.55	25.8	0.207	27	0
2	99	70	16	44	20.4	0.235	27	0
5	73	60	29.15	155.55	26.8	0.268	27	0
0	152	82	39	272	41.5	0.27	27	0
2	129	84	29.15	155.55	28	0.284	27	0
2	122	70	27	155.55	36.8	0.34	27	0
5	166	76	29.15	155.55	45.7	0.34	27	1
1	147	94	41	155.55	49.3	0.358	27	1
5	95	72	33	155.55	37.7	0.37	27	0
5	111	72	28	155.55	23.9	0.407	27	0
2	155	74	17	96	26.6	0.433	27	1
2	120	54	29.15	155.55	26.8	0.455	27	0
1	117	60	23	106	33.8	0.466	27	0
2	90	68	42	155.55	38.2	0.503	27	1
4	125	80	29.15	155.55	32.3	0.536	27	1
3	128	72	25	190	32.4	0.549	27	1
6	154	78	41	140	46.1	0.571	27	0
0	104	76	29.15	155.55	18.4	0.582	27	0
0	91	80	29.15	155.55	32.4	0.601	27	0
1	83	68	29.15	155.55	18.2	0.624	27	0
0	102	86	17	105	29.3	0.695	27	0
2	110	74	29	125	32.4	0.698	27	0
3	126	88	41	235	39.3	0.704	27	0
3	103	72	30	152	27.6	0.73	27	0
3	141	72.41	29.15	155.55	30	0.761	27	1
2	85	65	29.15	155.55	39.6	0.93	27	0
3	80	82	31	70	34.2	1.292	27	1
1	100	74	12	46	19.5	0.149	28	0
3	115	66	39	140	38.1	0.15	28	0
5	105	72	29	325	36.9	0.159	28	0
4	131	68	21	166	33.1	0.16	28	0
6	80	80	36	155.55	39.8	0.177	28	0
6	92	92	29.15	155.55	19.9	0.188	28	0
6	96	72.41	29.15	155.55	23.7	0.19	28	0
5	115	98	29.15	155.55	52.9	0.209	28	1
3	129	64	29	115	26.4	0.219	28	1
1	138	82	29.15	155.55	40.1	0.236	28	0
2	146	72.41	29.15	155.55	27.5	0.24	28	1
2	112	86	42	160	38.4	0.246	28	0
1	121	78	39	74	39	0.261	28	0
3	84	72	32	155.55	37.2	0.267	28	0
3	163	70	18	105	31.6	0.268	28	1
5	123	74	40	77	34.1	0.269	28	0
4	95	60	32	155.55	35.4	0.284	28	0
4	99	72	17	155.55	25.6	0.294	28	0
4	85	58	22	49	27.8	0.306	28	0
0	146	70	29.15	155.55	37.9	0.334	28	1
5	147	75	29.15	155.55	29.9	0.434	28	0
1	84	64	23	115	36.9	0.471	28	0
0	198	66	32	274	41.3	0.502	28	1
4	127	88	11	155	34.5	0.598	28	0

1	144	82	40	155.55	41.3	0.607	28	0
1	100	72	12	70	25.3	0.658	28	0
6	102	90	39	155.55	35.7	0.674	28	0
6	151	62	31	120	35.5	0.692	28	0
1	172	68	49	579	42.4	0.702	28	1
2	122	52	43	158	36.2	0.816	28	0
3	158	76	36	245	31.6	0.851	28	1
2	100	66	20	90	32.9	0.867	28	1
3	100	68	23	81	31.6	0.949	28	0
1	125	50	40	167	33.3	0.962	28	1
2	92	76	20	155.55	24.2	1.698	28	0
10	115	72.41	29.15	155.55	35.3	0.134	29	0
4	148	60	27	318	30.9	0.15	29	1
2	139	75	29.15	155.55	25.6	0.167	29	0
1	107	50	19	155.55	28.3	0.181	29	0
0	123	88	37	155.55	35.2	0.197	29	0
0	141	72.41	29.15	155.55	42.4	0.205	29	1
2	120	76	37	105	39.7	0.215	29	0
4	92	80	29.15	155.55	42.2	0.237	29	0
6	103	66	29.15	155.55	24.3	0.249	29	0
9	119	80	35	155.55	29	0.263	29	1
7	106	60	24	155.55	26.5	0.296	29	1
2	146	76	35	194	38.2	0.329	29	0
2	146	70	38	360	28	0.337	29	1
3	182	74	29.15	155.55	30.5	0.345	29	1
4	90	88	47	54	37.7	0.362	29	0
1	88	78	29	76	32	0.365	29	0
6	124	72	29.15	155.55	27.6	0.368	29	1
4	122	68	29.15	155.55	35	0.394	29	0
5	158	84	41	210	39.4	0.395	29	1
4	110	66	29.15	155.55	31.9	0.471	29	0
3	111	90	12	78	28.4	0.495	29	0
6	134	70	23	130	35.4	0.542	29	1
1	146	56	29.15	155.55	29.7	0.564	29	0
5	189	64	33	325	31.2	0.583	29	1
0	129	80	29.15	155.55	31.2	0.703	29	0
2	105	80	45	191	33.7	0.711	29	1
1	119	86	39	220	45.6	0.808	29	1
1	196	76	36	249	36.5	0.875	29	1
1	86	66	52	65	41.3	0.917	29	0
1	124	74	36	155.55	27.8	0.1	30	0
2	157	74	35	440	39.4	0.134	30	0
6	117	96	29.15	155.55	28.7	0.157	30	0
4	110	92	29.15	155.55	37.6	0.191	30	0
5	116	74	29.15	155.55	25.6	0.201	30	0
1	97	70	40	155.55	38.1	0.218	30	0
5	121	72	23	112	26.2	0.245	30	0
4	137	84	29.15	155.55	31.2	0.252	30	0
10	115	72.41	29.15	155.55	32.46	0.261	30	1
3	99	80	11	64	19.3	0.284	30	0
5	110	68	29.15	155.55	26	0.292	30	0

5	88	66	21	23	24.4	0.342	30	0
3	170	64	37	225	34.5	0.356	30	1
4	117	62	12	155.55	29.7	0.38	30	1
4	147	74	25	293	34.9	0.385	30	0
3	120	70	30	135	42.9	0.452	30	0
5	99	54	28	83	34	0.499	30	0
3	111	56	39	155.55	30.1	0.557	30	0
1	122	64	32	156	35.1	0.692	30	1
0	167	72.41	29.15	155.55	32.3	0.839	30	1
2	124	68	28	205	32.9	0.875	30	1
2	125	60	20	140	33.8	0.088	31	0
4	95	64	29.15	155.55	32	0.161	31	1
10	92	62	29.15	155.55	25.9	0.167	31	0
7	107	74	29.15	155.55	29.6	0.254	31	1
4	184	78	39	277	37	0.264	31	1
3	169	74	19	125	29.9	0.268	31	1
1	122	90	51	220	49.7	0.325	31	1
6	195	70	29.15	155.55	30.9	0.328	31	1
7	103	66	32	155.55	39.1	0.344	31	1
1	85	66	29	155.55	26.6	0.351	31	0
7	114	76	17	110	23.8	0.466	31	0
6	91	72.41	29.15	155.55	29.8	0.501	31	0
4	129	60	12	231	27.5	0.527	31	0
0	118	84	47	230	45.8	0.551	31	1
4	90	72.41	29.15	155.55	28	0.61	31	0
0	145	72.41	29.15	155.55	44.2	0.63	31	1
8	109	76	39	114	27.9	0.64	31	1
0	111	65	29.15	155.55	24.6	0.66	31	0
6	107	88	29.15	155.55	36.8	0.727	31	0
4	158	78	29.15	155.55	32.9	0.803	31	1
0	100	88	60	110	46.8	0.962	31	0
3	173	78	39	185	33.8	0.97	31	1
2	128	78	37	182	43.3	1.224	31	1
4	197	70	39	744	36.7	2.329	31	0
6	87	80	29.15	155.55	23.2	0.084	32	0
3	102	74	29.15	155.55	29.5	0.121	32	0
5	99	74	27	155.55	29	0.203	32	0
4	156	75	29.15	155.55	48.3	0.238	32	1
6	125	68	30	120	30	0.464	32	0
7	100	72.41	29.15	155.55	30	0.484	32	1
6	99	60	19	54	26.9	0.497	32	0
1	115	70	30	96	34.6	0.529	32	1
8	183	64	29.15	155.55	23.3	0.672	32	1
0	131	88	29.15	155.55	31.6	0.743	32	1
0	108	68	20	155.55	27.3	0.787	32	0
9	164	84	21	155.55	30.8	0.831	32	1
7	97	76	32	91	40.9	0.871	32	1
3	129	92	49	155	36.4	0.968	32	1
1	81	74	41	57	46.3	1.096	32	0
5	85	74	22	155.55	29	1.224	32	1
9	89	62	29.15	155.55	22.5	0.142	33	0

4	99	68	38	155.55	32.8	0.145	33	0
1	103	30	38	83	43.3	0.183	33	0
0	121	66	30	165	34.3	0.203	33	1
5	108	72	43	75	36.1	0.263	33	0
5	122	86	29.15	155.55	34.7	0.29	33	0
4	173	70	14	168	29.7	0.361	33	1
2	75	64	24	55	29.7	0.37	33	0
1	167	74	17	144	23.4	0.447	33	1
9	152	78	34	171	34.2	0.893	33	1
8	108	70	29.15	155.55	30.5	0.955	33	1
4	103	60	33	192	24	0.966	33	0
9	122	56	29.15	155.55	33.3	1.114	33	1
1	163	72	29.15	155.55	39	1.222	33	1
6	119	50	22	176	27.1	1.318	33	1
1	128	98	41	58	32	1.321	33	1
0	137	40	35	168	43.1	2.288	33	1
9	124	70	33	402	35.4	0.282	34	0
3	191	68	15	130	30.9	0.299	34	0
4	83	86	19	155.55	29.3	0.317	34	0
3	130	78	23	79	28.4	0.323	34	1
4	123	80	15	176	32	0.443	34	0
10	168	74	29.15	155.55	38	0.537	34	1
5	155	84	44	545	38.7	0.619	34	0
4	120	68	29.15	155.55	29.6	0.709	34	0
7	114	64	29.15	155.55	27.4	0.732	34	1
6	123	72	45	230	33.6	0.733	34	0
7	187	50	33	392	33.9	0.826	34	1
8	107	80	29.15	155.55	24.6	0.856	34	0
10	115	98	29.15	155.55	24	1.022	34	0
2	106	64	35	119	30.5	1.4	34	0
5	77	82	41	42	35.8	0.156	35	0
4	123	62	29.15	155.55	32	0.226	35	1
5	136	84	41	88	35	0.286	35	1
11	85	74	29.15	155.55	30.1	0.3	35	0
0	180	90	26	90	36.5	0.314	35	1
3	158	70	30	328	35.5	0.344	35	1
11	138	76	29.15	155.55	33.2	0.42	35	0
5	116	74	29	155.55	32.3	0.66	35	1
6	108	44	20	130	24	0.813	35	0
0	93	100	39	72	43.4	1.021	35	0
6	102	82	29.15	155.55	30.8	0.18	36	1
4	183	72.41	29.15	155.55	28.4	0.212	36	1
10	133	68	29.15	155.55	27	0.245	36	0
0	124	70	20	155.55	27.4	0.254	36	1
9	112	82	32	175	34.2	0.26	36	1
4	151	90	38	155.55	29.7	0.294	36	0
7	152	88	44	155.55	50	0.337	36	1
7	159	66	29.15	155.55	30.4	0.383	36	1
3	187	70	22	200	36.4	0.408	36	1
8	151	78	32	210	42.9	0.516	36	1
5	44	62	29.15	155.55	25	0.587	36	0

3	174	58	22	194	32.9	0.593	36	1
1	95	74	21	73	25.9	0.673	36	0
8	179	72	42	130	32.7	0.719	36	1
7	83	78	26	71	29.3	0.767	36	0
1	90	68	8	155.55	24.5	1.138	36	0
6	105	70	32	68	30.8	0.122	37	0
7	124	70	33	215	25.5	0.161	37	0
10	179	70	29.15	155.55	35.1	0.2	37	0
3	150	76	29.15	155.55	21	0.207	37	0
7	119	72.41	29.15	155.55	25.2	0.209	37	0
5	137	108	29.15	155.55	48.8	0.227	37	1
5	88	78	30	155.55	27.6	0.258	37	0
7	133	88	15	155	32.4	0.262	37	0
4	144	58	28	140	29.5	0.287	37	0
4	154	72	29	126	31.3	0.338	37	0
5	121.69	80	32	155.55	41	0.346	37	1
8	186	90	35	225	34.5	0.423	37	1
4	114	65	29.15	155.55	21.9	0.432	37	0
4	116	72	12	87	22.1	0.463	37	0
4	144	82	32	155.55	38.5	0.554	37	1
4	189	110	31	155.55	28.5	0.68	37	0
7	133	84	29.15	155.55	40.2	0.696	37	0
5	130	82	29.15	155.55	39.1	0.956	37	1
1	128	88	39	110	36.5	1.057	37	1
1	173	74	29.15	155.55	36.8	0.088	38	1
8	120	72.41	29.15	155.55	30	0.183	38	1
14	175	62	30	155.55	33.6	0.212	38	1
5	124	74	29.15	155.55	34	0.22	38	1
4	112	78	40	155.55	39.4	0.236	38	0
10	75	82	29.15	155.55	33.3	0.263	38	0
9	72	78	25	155.55	31.6	0.28	38	0
5	106	82	30	155.55	39.5	0.286	38	0
1	181	64	30	180	34.1	0.328	38	1
5	117	92	29.15	155.55	34.1	0.337	38	0
10	129	62	36	155.55	41.2	0.441	38	1
13	104	72	29.15	155.55	31.2	0.465	38	1
3	89	74	16	85	30.4	0.551	38	0
12	151	70	40	271	41.8	0.742	38	1
0	109	88	30	155.55	32.5	0.855	38	1
10	101	86	37	155.55	45.6	1.136	38	1
8	126	74	38	75	25.9	0.162	39	0
3	78	70	29.15	155.55	32.5	0.27	39	0
8	133	72	29.15	155.55	32.9	0.27	39	1
10	129	76	28	122	35.9	0.28	39	0
7	137	90	41	155.55	32	0.391	39	0
8	84	74	31	155.55	38.3	0.457	39	0
7	160	54	32	175	30.5	0.588	39	1
8	74	70	40	49	35.3	0.705	39	0
6	154	74	32	193	29.3	0.839	39	0
3	96	56	34	115	24.7	0.944	39	0
13	153	88	37	140	40.6	1.174	39	0

8	197	74	29.15	155.55	25.9	1.191	39	1
10	111	70	27	155.55	27.5	0.141	40	1
3	96	78	39	155.55	37.3	0.238	40	0
4	141	74	29.15	155.55	27.6	0.244	40	0
6	115	60	39	155.55	33.7	0.245	40	1
3	122	78	29.15	155.55	23	0.254	40	0
6	144	72	27	228	33.9	0.255	40	0
7	159	64	29.15	155.55	27.4	0.294	40	0
9	123	70	44	94	33.1	0.374	40	0
1	117	88	24	145	34.5	0.403	40	1
5	126	78	27	22	29.6	0.439	40	0
11	135	72.41	29.15	155.55	52.3	0.578	40	1
9	145	80	46	130	37.9	0.637	40	1
7	168	88	42	321	38.2	0.787	40	1
9	57	80	37	155.55	32.8	0.096	41	0
8	143	66	29.15	155.55	34.9	0.129	41	1
5	168	64	29.15	155.55	32.9	0.135	41	1
13	76	60	29.15	155.55	32.8	0.18	41	0
10	125	70	26	115	31.1	0.205	41	1
12	140	85	33	155.55	37.4	0.244	41	0
7	187	68	39	304	37.7	0.254	41	1
10	122	68	29.15	155.55	31.2	0.258	41	0
5	112	66	29.15	155.55	37.8	0.261	41	1
1	180	72.41	29.15	155.55	43.3	0.282	41	1
2	129	72.41	29.15	155.55	38.5	0.304	41	0
6	80	66	30	155.55	26.2	0.313	41	0
7	178	84	29.15	155.55	39.9	0.331	41	1
7	184	84	33	155.55	35.5	0.355	41	1
7	62	78	29.15	155.55	32.6	0.391	41	0
0	189	104	25	155.55	34.3	0.435	41	1
7	196	90	29.15	155.55	39.8	0.451	41	1
6	104	74	18	156	29.9	0.722	41	1
6	121.69	68	41	155.55	39	0.727	41	1
7	94	64	25	79	33.3	0.738	41	0
7	194	68	28	155.55	35.9	0.745	41	1
1	120	80	48	200	38.9	1.162	41	0
11	103	68	40	155.55	46.2	0.126	42	0
8	85	55	20	155.55	24.4	0.136	42	0
8	100	76	29.15	155.55	38.7	0.19	42	0
5	117	86	30	105	39.1	0.251	42	0
7	114	66	29.15	155.55	32.8	0.258	42	1
11	136	84	35	130	28.3	0.26	42	1
7	81	78	40	48	46.7	0.261	42	0
10	108	66	29.15	155.55	32.4	0.272	42	1
2	89	90	30	155.55	33.5	0.292	42	0
1	102	74	29.15	155.55	39.5	0.293	42	1
1	149	68	29	127	29.3	0.349	42	1
9	106	52	29.15	155.55	31.2	0.38	42	0
6	85	78	29.15	155.55	31.2	0.382	42	0
1	100	66	29	196	32	0.444	42	0
13	126	90	29.15	155.55	43.4	0.583	42	1

8	65	72	23	155.55	32	0.6	42	0
7	150	66	42	342	34.7	0.718	42	0
9	156	86	28	155	34.3	1.189	42	1
7	142	90	24	480	30.4	0.128	43	1
8	188	78	29.15	155.55	47.9	0.137	43	1
15	136	70	32	110	37.1	0.153	43	1
8	167	106	46	231	37.6	0.165	43	1
1	95	82	25	180	35	0.233	43	1
7	147	76	29.15	155.55	39.4	0.257	43	1
9	170	74	31	155.55	44	0.403	43	1
6	98	58	33	190	34	0.43	43	0
7	129	68	49	125	38.5	0.439	43	1
8	100	74	40	215	39.4	0.661	43	1
13	152	90	33	29	26.8	0.731	43	1
5	96	74	18	67	33.6	0.997	43	0
7	109	80	31	155.55	35.9	1.127	43	1
12	106	80	29.15	155.55	23.6	0.137	44	0
13	158	114	29.15	155.55	42.3	0.257	44	1
5	115	76	29.15	155.55	31.2	0.343	44	1
3	132	80	29.15	155.55	34.4	0.402	44	1
13	129	72.41	30	155.55	39.9	0.569	44	1
12	92	62	7	258	27.6	0.926	44	1
0	117	72.41	29.15	155.55	33.8	0.932	44	0
0	146	82	29.15	155.55	40.5	1.781	44	0
5	128	80	29.15	155.55	34.6	0.144	45	0
9	164	78	29.15	155.55	32.8	0.148	45	1
9	154	78	30	100	30.9	0.164	45	0
13	106	72	54	155.55	36.6	0.178	45	0
7	102	74	40	105	37.2	0.204	45	0
1	133	102	28	140	32.8	0.234	45	1
8	105	100	36	155.55	43.3	0.239	45	1
1	111	94	29.15	155.55	32.8	0.265	45	0
8	154	78	32	155.55	32.4	0.443	45	1
10	122	78	31	155.55	27.6	0.512	45	0
9	130	70	29.15	155.55	34.2	0.652	45	1
9	140	94	29.15	155.55	32.7	0.734	45	1
11	111	84	40	155.55	46.8	0.925	45	1
4	125	70	18	122	28.9	1.144	45	1
6	183	94	29.15	155.55	40.8	1.461	45	0
6	92	62	32	126	32	0.085	46	0
0	67	76	29.15	155.55	45.3	0.194	46	0
0	105	90	29.15	155.55	29.6	0.197	46	0
6	134	80	37	370	46.2	0.238	46	1
3	61	82	28	155.55	34.4	0.243	46	0
12	84	72	31	155.55	29.7	0.297	46	1
1	144	82	46	180	46.1	0.335	46	1
4	115	72	29.15	155.55	28.9	0.376	46	1
14	100	78	25	184	36.6	0.412	46	1
12	100	84	33	105	30	0.488	46	0
8	155	62	26	495	34	0.543	46	1
9	102	76	37	155.55	32.9	0.665	46	1

8	118	72	19	155.55	23.1	1.476	46	0
7	161	86	29.15	155.55	30.4	0.165	47	1
5	143	78	29.15	155.55	45	0.19	47	0
10	68	106	23	49	35.5	0.285	47	0
10	161	68	23	132	25.5	0.326	47	1
1	126	60	29.15	155.55	30.1	0.349	47	1
17	163	72	41	114	40.9	0.817	47	1
5	104	74	29.15	155.55	28.8	0.153	48	0
7	106	92	18	155.55	22.7	0.235	48	0
12	88	74	40	54	35.3	0.378	48	0
9	120	72	22	56	20.8	0.733	48	0
11	120	80	37	150	42.3	0.785	48	1
9	165	88	29.15	155.55	30.4	0.302	49	1
8	126	88	36	108	38.5	0.349	49	0
6	125	78	31	155.55	27.6	0.565	49	1
6	165	68	26	168	33.6	0.631	49	0
9	184	85	15	155.55	30	1.213	49	1
6	162	62	29.15	155.55	24.3	0.178	50	1
6	147	80	29.15	155.55	29.5	0.178	50	1
7	136	90	29.15	155.55	29.9	0.21	50	0
1	164	82	43	67	32.8	0.341	50	0
8	99	84	29.15	155.55	35.4	0.388	50	0
11	138	74	26	144	36.1	0.557	50	1
6	148	72	35	155.55	33.6	0.627	50	1
9	112	82	24	155.55	28.2	1.282	50	1
11	127	106	29.15	155.55	39	0.19	51	0
11	143	94	33	146	36.6	0.254	51	1
7	125	86	29.15	155.55	37.6	0.304	51	0
7	181	84	21	192	35.9	0.586	51	1
5	166	72	19	175	25.8	0.587	51	1
7	136	74	26	135	26	0.647	51	0
10	148	84	48	237	37.6	1.001	51	1
11	155	76	28	150	33.3	1.353	51	1
5	162	104	29.15	155.55	37.7	0.151	52	1
13	106	70	29.15	155.55	34.2	0.251	52	0
0	123	72	29.15	155.55	36.3	0.258	52	1
2	108	80	29.15	155.55	27	0.259	52	1
5	97	76	27	155.55	35.6	0.378	52	1
8	124	76	24	600	28.7	0.687	52	1
1	168	88	29	155.55	35	0.905	52	1
3	176	86	27	156	33.3	1.154	52	1
2	197	70	45	543	30.5	0.158	53	1
9	156	86	29.15	155.55	24.8	0.23	53	1
2	105	75	29.15	155.55	23.3	0.56	53	0
9	145	88	34	165	30.3	0.771	53	1
5	187	76	27	207	43.6	1.034	53	1
6	125	76	29.15	155.55	33.8	0.121	54	1
10	162	84	29.15	155.55	27.7	0.182	54	0
8	125	96	29.15	155.55	32.46	0.232	54	1
7	150	78	29	126	35.2	0.692	54	1
9	171	110	24	240	45.4	0.721	54	1

2	109	92	29.15	155.55	42.7	0.845	54	0
6	137	61	29.15	155.55	24.2	0.151	55	0
7	195	70	33	145	25.1	0.163	55	1
3	128	78	29.15	155.55	21.1	0.268	55	0
6	103	72	32	190	37.7	0.324	55	0
10	94	72	18	155.55	23.1	0.595	56	0
10	90	85	32	155.55	34.9	0.825	56	1
4	111	72	47	207	37.1	1.39	56	1
13	145	82	19	110	22.2	0.245	57	0
8	95	72	29.15	155.55	36.8	0.485	57	0
8	196	76	29	280	37.5	0.605	57	1
5	114	74	29.15	155.55	24.9	0.744	57	0
10	139	80	29.15	155.55	27.1	1.441	57	0
9	91	68	29.15	155.55	24.2	0.2	58	0
8	110	76	29.15	155.55	27.8	0.237	58	0
5	144	82	26	285	32	0.452	58	1
8	176	90	34	300	33.7	0.467	58	1
12	140	82	43	325	39.2	0.528	58	1
8	112	72	29.15	155.55	23.6	0.84	58	0
0	173	78	32	265	46.5	1.159	58	0
6	194	78	29.15	155.55	23.5	0.129	59	1
0	137	84	27	155.55	27.3	0.231	59	0
1	189	60	23	846	30.1	0.398	59	1
7	179	95	31	155.55	34.2	0.164	60	0
4	134	72	29.15	155.55	23.8	0.277	60	1
5	109	75	26	155.55	36	0.546	60	0
6	129	90	7	326	19.6	0.582	60	0
8	181	68	36	495	30.1	0.615	60	1
4	146	92	29.15	155.55	31.2	0.539	61	1
7	142	60	33	190	28.8	0.687	61	0
12	121	78	17	155.55	26.5	0.259	62	0
2	197	70	99	155.55	34.7	0.575	62	1
1	135	54	29.15	155.55	26.7	0.687	62	0
0	105	84	29.15	155.55	27.9	0.741	62	1
10	101	76	48	180	32.9	0.171	63	0
3	142	80	15	155.55	32.4	0.2	63	0
5	158	70	29.15	155.55	29.8	0.207	63	0
4	132	86	31	155.55	28	0.419	63	0
8	120	78	29.15	155.55	25	0.409	64	0
5	147	78	29.15	155.55	33.7	0.218	65	0
0	161	50	29.15	155.55	21.9	0.254	65	0
5	103	108	37	155.55	39.2	0.305	65	0
6	114	88	29.15	155.55	27.8	0.247	66	0
6	190	92	29.15	155.55	35.5	0.278	66	1
6	166	74	29.15	155.55	26.6	0.304	66	0
2	158	90	29.15	155.55	31.6	0.805	66	1
4	146	78	29.15	155.55	38.5	0.52	67	1
8	194	80	29.15	155.55	26.1	0.551	67	0
0	57	60	29.15	155.55	21.7	0.735	67	0
8	91	82	29.15	155.55	35.6	0.587	68	0
5	132	80	29.15	155.55	26.8	0.186	69	0

5	136	82	29.15	155.55	32.46	0.64	69	0
4	145	82	18	155.55	32.5	0.235	70	1
2	119	72.41	29.15	155.55	19.6	0.832	72	0
9	134	74	33	60	25.9	0.46	81	0
