# Supplementary material for: Improved Functional Causal Likelihood-Based Causal Discovery Method for Diabetes Risk Factors
Source: Comput Math Methods Med. 2021 May 14;2021:5552085. doi: 10.1155/2021/5552085 (PMC8143882; doi:10.1155/2021/5552085)
Supplement: Supplementary Materials — See Datasets S1–S2, Source Code S3, and Editorial Certificate S4 in the Supplementary Material for simulation experiments. [file 5552085.f1.zip › S3.pdf]

Caption

This is the article experiment source code; The experimental data are from the website, see the detailed description of the experimental section of the paper; Adopt R language programming environment.

```
#####################################################################################
install.packages("SELF")
library(SELF)
library(psych)
data <- read.csv("C:/Users/Administrator/Desktop/diabetes/diabetes1.csv") #input 768 dataset that has been preprocessed.
data2<- read.csv("C:/Users/Administrator/Desktop/diabetes/diabetes1.csv") #input 2000 dataset that has been preprocessed.
ldata <- read.csv("C:/Users/Administrator/Desktop/diabetes/diabetes1.csv")
2data <- read.csv("C:/Users/Administrator/Desktop/diabetes/diabetes2.csv")
class(data)
head(data)
class(data2)
head(data2)
#########################################M=768 dataset
D <- data[-1,]
P <- D[,1]
G <- D[,2]
B <- D[,3]
S <- D[,4]
I <- D[,5]
B1 <- D[,6]
A <- D[,7]
D1 <- D[,8]
#######################################M=2000 dataset
D2 <- data2[-1,]
P1 <- D2[,1]
G1 <- D2[,2]
B2 <- D2[,3]
S 1<- D2[,4]
I1 <- D2[,5]
B3 <- D2[,6]
A 1<- D2[,7]
D3 <- D2[,8]
########################################Scatter plos, bar charts and correlation coefficients
pairs.panels(ldata[,1:8],lm=FALSE)
pairs.panels(2data[,1:8],lm=FALSE)
######################################### FCL-based Casual discovery
data1 <- data.frame(P,G,B,S,I,B1,A,D1)
data3 <- data.frame(P1,G1,B2,S1,I1,B3,A1,D3)
```

```
fhc(data1,booster = "lm",score_type = "bic") # casual discovery of M=768 dataset
fhc(data3,booster = "lm",score_type = "bic")# casual discovery of M=2000 dataset
# IFCL-based Casual discovery
require(stats)
trace("fhc",edit = TRUE) #insert the the threshold to improve the casual discovery method
fhc(data1,booster = "lm",score_type = "bic") # the improved casual discovery of M=768 dataset
fhc(data3,booster = "lm",score_type = "bic")# the improved casual discovery of M=2000 dataset
```
