# Supplementary material for: Improved Functional Causal Likelihood-Based Causal Discovery Method for Diabetes Risk Factors
Source: Comput Math Methods Med. 2021 May 14;2021:5552085. doi: 10.1155/2021/5552085 (PMC8143882; doi:10.1155/2021/5552085)
Supplement: Supplementary Materials — See Datasets S1–S2, Source Code S3, and Editorial Certificate S4 in the Supplementary Material for simulation experiments. [file 5552085.f1.zip › S4.pdf]

Editing Certificate

This document certifies that the manuscript listed below has been edited to ensure language and grammar accuracy and is error free in these aspects. The logical presentation of ideas and the structure of the paper were also checked during the editing process. The edit was performed by professional editors at Editage, a division of Cactus Communications. The author's core research ideas were not altered in any way during the editing process. The quality of the edit has been guaranteed, with the assumption that our suggested changes have been accepted and the text has not been further altered without the knowledge of our editors.

MANUSCRIPT TITLE

Improved functional causal likelihood-based causal discovery method for diabetes risk factors

AUTHORS

Xiue Gao, Wenxue Xie, Zumin Wang, Bo Chen, Shengbin Zhou

ISSUED ON

April 19, 2021

JOB CODE

WEUEX_1_2

THE EDITAGE Promise QUALITY GUARANTEE

Vikas Narang

Vikas Narang
Chief Operating Officer - Editage

Editage, a brand of Cactus Communications, offers professional English language editing and publication support services to authors engaged in over 1300 areas of research. Through its community of experienced editors, which includes doctors, engineers, published scientists, and researchers with peer review experience, Editage has successfully helped authors get published in internationally reputed journals. Authors who work with Editage are guaranteed excellent language quality and timely delivery.

GLOBAL : (833) 979-0061 | request@editage.com

CHINA : 02158209795 | fabiao@editage.cn

CACTUS

impact.science

researcher.life

lifesciences.cactusglobal.com
